# Supplementary figures and images for: Seroprevalence of anti-SARS-CoV-2 antibodies in a cohort of New York City metro blood donors using multiple SARS-CoV-2 serological assays: Implications for controlling the epidemic and “Reopening”
Source: PLoS One. 2021 Apr 28;16(4):e0250319. doi: 10.1371/journal.pone.0250319 (PMC8081167; doi:10.1371/journal.pone.0250319)

**
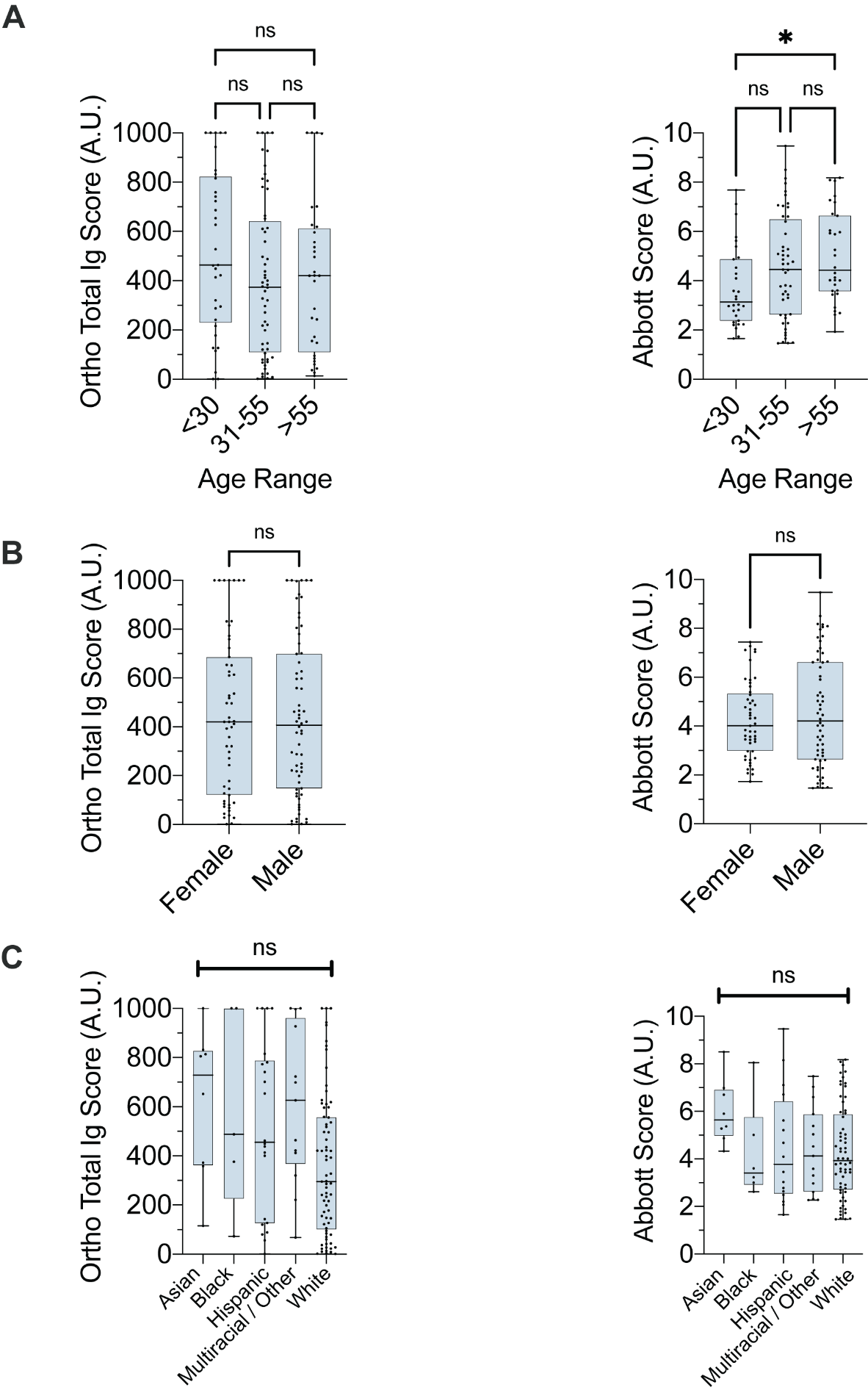
**

Supplement: S1 Fig — A; Distribution of Ortho Total Ig (left) or Abbott IgG (right) HTSA scores among seropositive blood donors by age range groups. N = 129, one-way-ANOVA (Kruskal-Wallace test), * p < 0.05. B; Distribution of Ortho Total Ig (left) or Abbott IgG (right) HTSA scores among seropositive blood donors by sex. N = 129, student’s T test (two-tailed). C; Distribution of Ortho Total Ig (left) or Abbott IgG (right) HTSA scores among seropositive blood donors by age reported ethnicity. N = 129, one-way-ANOVA (Kruskal-Wallace test). (DOCX) [file pone.0250319.s001.docx]
